# microRNA-222 Targeting PTEN Promotes Neurite Outgrowth from Adult Dorsal Root Ganglion Neurons following Sciatic Nerve Transection

**DOI:** 10.1371/journal.pone.0044768

**Published:** 2012-09-13

**Authors:** Songlin Zhou, Dingding Shen, Yongjun Wang, Leilei Gong, Xiaoyan Tang, Bin Yu, Xiaosong Gu, Fei Ding

**Affiliations:** 1 Jiangsu Key Laboratory of Neuroregeneration, Nantong University, Nantong, China; 2 Key Lab of Systems Biology, Shanghai Institutes for Biological Sciences, Chinese Academy of Sciences, Shanghai, China; Schepens Eye Research Institute, Harvard Medical School, United States of America

## Abstract

Dorsal root ganglia (DRG) neurons spontaneously undergo neurite growth after nerve injury. MicroRNAs (miRNAs), as small, non-coding RNAs, negatively regulate gene expression in a variety of biological processes. The roles of miRNAs in the regulation of responses of DRG neurons to injury stimuli, however, are not fully understood. Here, microarray analysis was performed to profile the miRNAs in L4-L6 DRGs following rat sciatic nerve transection. The 26 known miRNAs were differentially expressed at 0, 1, 4, 7, 14 d post injury, and the potential targets of the miRNAs were involved in nerve regeneration, as analyzed by bioinformatics. Among the 26 miRNAs, microRNA-222 (miR-222) was our research focus because its increased expression promoted neurite outgrowth while it silencing by miR-222 inhibitor reduced neurite outgrowth. Knockdown experiments confirmed that phosphatase and tensin homolog deleted on chromosome 10 (PTEN), a major inhibitor of nerve regeneration, was a direct target of miR-222 in DRG neurons. In addition, we found that miR-222 might regulate the phosphorylation of cAMP response element binding protein (CREB) through PTEN, and c-Jun activation might enhance the miR-222 expression. Collectively, our data suggest that miR-222 could regulate neurite outgrowth from DRG neurons by targeting PTEN.

## Introduction

Upon injury to peripheral nerves, the proximal nerve stump will spontaneously regenerate due to activation of the intrinsic growth capacity of neurons. The sciatic nerve, comprising a mixed population of motor and sensory axons, is a commonly used model in nerve regeneration studies. The sensory neurons extending into the sciatic nerve are located in the L4-L6 dorsal root ganglia (DRGs). Once primary sensory neurons are primed by peripheral axonal injury, they grow more rapidly in response to a subsequent lesion, which has been known as the conditioning effect [1], [2]. Peripheral axonal injury triggers the intrinsic growth of DRG neurons, “conditions” the neurons to grow extensively *in vitro* [3], and facilitates peripheral nerve regeneration *in vivo* [4]. The “conditioning injuries” also permit DRG neuronal growth on normally nonpermissive central myelin substrates, such as myelin-associated glycoprotein (MAG) or myelin [5], and enable the central axons of DRG neurons to regenerate into and beyond the injury site in the inhibitory environment of the spinal cord [6]. Strikingly, regeneration is enhanced in axons re-injured 1–2 weeks after a conditioning lesion [7]. In other words, peripheral “conditioning” lesion indeed switches DRG neurons from a transmitter to a regenerative state, indicating that the robust response of peripheral axons to injury is not merely a “default” state, but results from activation of injury signals, which travel retrogradely from the peripheral lesion site to the cell body and enhance the intrinsic growth capacity of the neurons [8]. It is generally known that phosphatase and tensin homolog deleted on chromosome 10 (PTEN), an endogenous inhibitor of the phosphoinositide 3-kinase (PI_3_K) pathway, is important for central axon growth [9]. PTEN inhibition facilitates the intrinsic regenerative outgrowth of adult peripheral axons, and PTEN might act as an intrinsic brake on the regenerative outgrowth [10]. Now, the cyclic adenosine monophosphate (cAMP)-protein kinase A (PKA)-cAMP response element binding protein (CREB) signaling and the c-Jun transcription factor have been known to activate the intrinsic growth capacity mainly at the transcriptional level after peripheral nerve injury [11], [12]. The detailed signaling pathways responsible for the intrinsic regeneration, however, remain to be further investigated.

microRNAs (miRNAs), a class of approximately 22 nucleotide non-coding RNA molecules, negatively regulate the expression of a wide variety of genes mainly through a direct interaction with the 3′-untranslated regions (3′-UTRs) of their corresponding mRNA targets [13]. It has been estimated that miRNAs regulate up to 60% of the total human genes at the post-transcriptional level [14], indicating that miRNAs play pivotal roles in physiological and pathological processes. The importance of miRNA in neural development and neurodegeneration is starting to be recognized [15]-[17], and miRNA expression profiles have been found to be significantly altered in the spinal cord injury model of adult rats [18], [19]. We also performed microarray and deep sequencing to show that abnormal expressions of miRNA in DRGs might be involved in molecular mechanisms of nerve regeneration after sciatic nerve transection [20], [21]. These results are consistent with the previous findings that Dicer-mediated miRNAs pathway is required for effective and timely regeneration of peripheral nerves *in vivo* or for regenerative axon growth *in vitro* [22], [23]. In particular, the role of miRNA in neurite outgrowth from DRG neurons has recently been shown by two independent studies [24], [25]. While the post-injury time points chosen for analyzing miRNA expression profiles are different among studies, each study shows that axotomy-induced miRNAs could regulate neurite growth. For example, miR-21 promotes neurite outgrowth by directly down-regulating Sprouty2 expression; while miRNA-145 inhibites neurite outgrowth by inhibiting Robo2 expression. To date, however, no reports are available on profiling miRNAs following peripheral nerve injury to elucidate, in a systematic way, vital roles of miRNAs in controlling the phenotypic changes of DRG neurons throughout the time period of nerve regeneration.

In this study we aimed to investigate the role of miRNAs in regulating gene expression and functions of DRG neurons during peripheral nerve regeneration. Our results indicated that miR-222 definitely promoted neurite outgrowth by targeting PTEN and increasing intracellular phosphorylated CREB (pCREB) levels.

## Results

### miRNA Expression Profiling in DRGs Following Sciatic Nerve Injury

**Figure 1:**
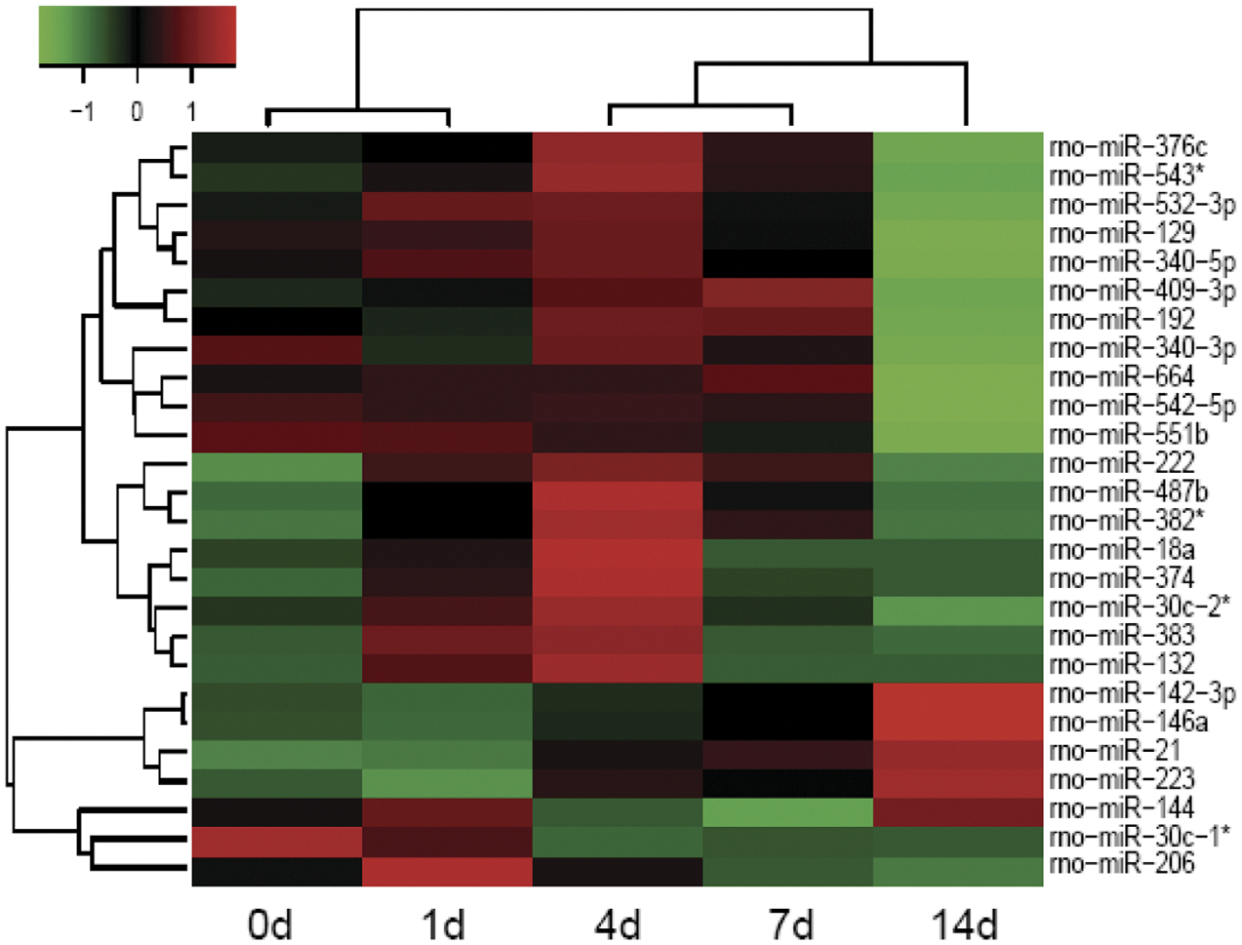
Heatmap and cluster dendrogram of differentially expressed 26 miRNAs that showed significant changes at four time points after nerve transection. The color scale shown on the top illustrates the relative expression level of the indicated miRNA across all samples: red denotes expression >0 and green denotes expression <0.

Agilent miRNA microarray plus Random Variation Model screening were used to determine the expression profile of miRNAs in DRGs after sciatic nerve transection. A total of 26 miRNAs showed differential expressions at 0, 1, 4, 7, and 14 d post injury. The 5 time points were chosen to investigate the miRNA expression profile because the active nerve regeneration could be observed over this time period. Hierarchical cluster analysis indicated that there was a certain similarity in the miRNA profile between day 0 (control) and day 1 groups or between day 4, 7, and 14 groups, respectively, but the similar miRNA expressions at days 0 and 1 were significantly different from those at days 4, 7, and 14 (Figure 1). These results are in line with previous observations [10], [26].

Then, we searched for the putative targets of the 26 miRNAs using TargetScan and miRanda database. The intersection of these two datasets was used for predicting the target genes of the 26 differentially expressed miRNAs, thus yielding 638 putative miRNA targets, which were integrated with differentially expressed mRNAs to obtain 38 potential targets. In order to know credible biological functions, we conducted the Gene Ontology (GO) analyses regarding the intersected genes. The GO terms with the highest enriched scores and most significant *P* values for 638 putative targets are listed in Table 1. The most significant GO functions were related to the cell phenotype modulation, including neuron differentiation, regulation of cellular component movement, negative regulation of programmed cell death, cellular localization. The GO terms for 38 potential targets are also listed (Table 1). Strikingly, the most significant GO functions were associated with the structural integrity of the axonal plasma membrane and electrical properties of injured nerves, such as glutamine transport, sodium ion transport, regulation of transmembrane transporter activity.

**Table 1:** Gene ontology analysis for putative miRNA targets (638 gene).

GO ID	GO terms	*P* value	qFDR
GO:0023034	intracellular signaling pathway	4.41E-08	7.95E-06
GO:0030182	neuron differentiation	4.44E-08	7.95E-06
GO:0045944	positive regulation of transcription from RNA polymerase II promoter	6.70E-08	1.14E-05
GO:0065008	regulation of biological quality	2.56E-07	3.98E-05
GO:0051270	regulation of cellular component movement	2.08E-06	0.000267
GO:0045597	positive regulation of cell differentiation	2.56E-06	0.0003
GO:0051641	cellular localization	2.85E-06	0.0003
GO:0008104	protein localization	3.95E-06	0.0004
GO:0043069	negative regulation of programmed cell death	4.88E-06	0.0005
GO:0000904	cell morphogenesis involved in differentiation	6.62E-06	0.0007
for overlapping of putative miRNA targets and differentially expressed genes (38 gene)			
GO:0006868	glutamine transport	6.56E-06	0.003
GO:0006814	sodium ion transport	1.81E-05	0.005
GO:0007275	multicellular organismal development	1.94E-05	0.005
GO:0009607	response to biotic stimulus	0.000144	0.018
GO:0022898	regulation of transmembrane transporter activity	0.0002	0.018

### Functional Investigation of miR-222

**Figure 2:**
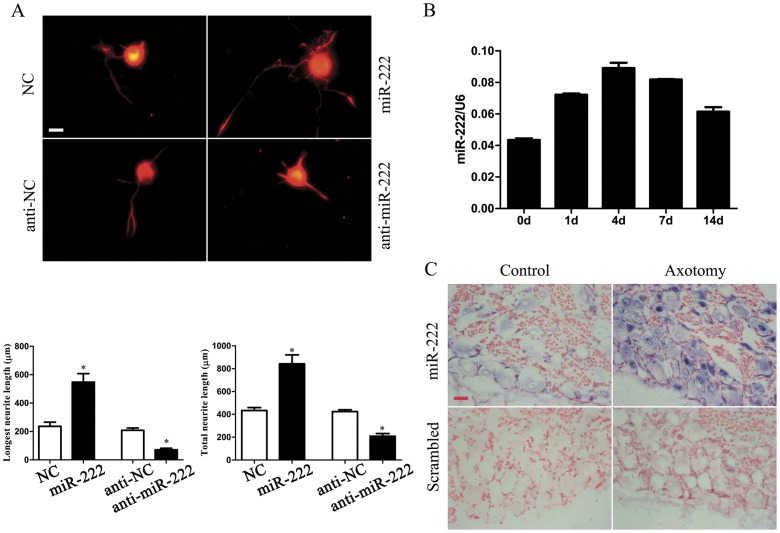
miR-222 increased neurite outgrowth from DRG neurons. A. Representative immunocytochemistry with anti-NF-200 (red) for DRG neurons, which were transfected with miR-222 mimics (miR-222), mimics control (NC), or miR-222 inhibitor (anti-miR-222), inhibitor control (anti-NC), respectively, to observe neurite outgrowth. Scale bar, 30 µm. Histogram shows comparison in the mean longest neurite length and mean total neurite length (per neuron) between transfected cultures and controls, as observed at 72 h after transfection. *P<0.05 vs NC or anti-NC. B. qRT-PCR validation of the miR-222 level in DRGs at different time points following sciatic nerve transection. Expression of miR-222 was normalized to that of RNU6B. C. *In situ* hybridization with miR-222 and control scrambled probes showed up-regulation of miR-222 following axotomy and miR-222 (blue) localization in DRGs. Scale bar, 30 µm.

To test the involvement of 26 dys-regulated miRNAs in cellular functions, adult DRG neurons were transfected with miRNA mimics, miRNA inhibitor, or non-targeting negative controls (NC), respectively, followed by neurite outgrowth assay. Using the miRNA application protocol, we observed that the transfection efficiency was nearly 100% (data not shown). After testing each of the 26 miRNAs, we found that transfection of miR-222 mimics (miR-222) significantly increased the outgrowth ability of DRG neurons by approximately 2-folds as compared to NC cells. In contrast, silencing of miR-222 in neurons by its inhibitor was noted to markedly impair neurite outgrowth (Figure 2A). Quantification of neurite outgrowth of neurons indicated that transfection of miR-222 significantly increased the mean longest neurite length as compared to the NC cells (547±85.1 µm vs 237±41 µm, *P*<0.05), and increased the mean total neurite length (per neuron) as compared to the NC cells (843±111.7 µm vs 433±36.8 µm, *P*<0.05) at 72 h after nerve transfection. These data suggested that overexpression of miR-222 increased neurite elongation of DRG neurons and promoted their regeneration.

Quantitative real-time polymerase chain reaction (qRT-PCR) provided further evidence for the above-mentioned microarray data (Figure 2B). *In situ* hybridization experiments performed at 4 d post injury showed that the up-regulation of miR-222 occurred mainly in DRG neurons, while miR-222 was not or weakly expressed in Schwann cells (Figure 2C).

### Down-regulation of PTEN Expression by miR-222

**Figure 3:**
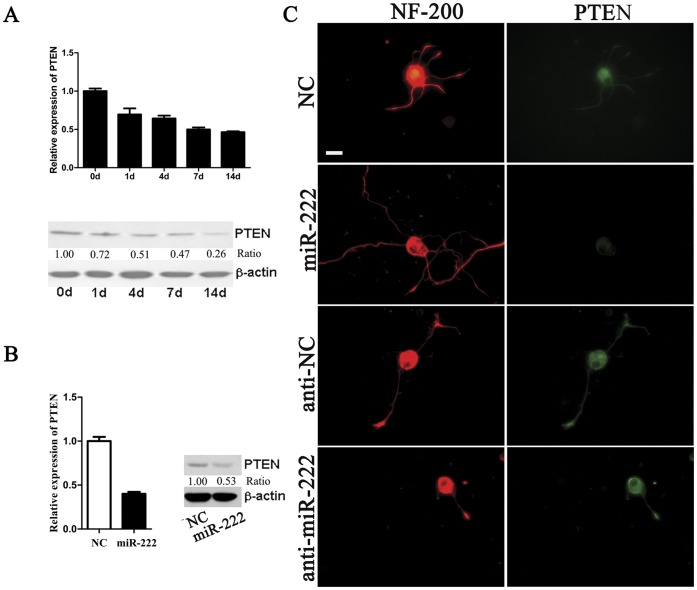
PTEN was a direct target of miR-222. A. The PTEN expression at the mRNA (histogram) and protein level (representative Western blot image) was gradually reduced until day 14 post injury, as compared to day 0 (control), and β-actin served as an internal control. B. The mRNA or protein level of PTEN was down-regulated by transfection with miR-222 mimics (miR-222) as compared with that of mimics control (NC) in DRG neurons. C. Representative micrographs following immunocytochemistry with anti-NF-200 (red) and anti-PTEN (green) showed that transfection with miR-222 reduced PTEN in the soma of DRG neurons as compared with that of NC in DRG neurons, while transfection with miR-222 inhibitor (anti-miR-222) resulted in accumulation of PTEN in the neurite end of DRG neurons as compared with that of inhibitor control (anti-NC) in DRG neurons. Scale bar, 30 µm.

PTEN has been identified as a mediator of miR-222 in regulation of cell growth capacities of several cancer cell types [27], [28]. Driven by this finding, we examined the mRNA and protein levels of PTEN, which were gradually decreased in adult DRGs at different time points post injury (Figure 3A). The PTEN expression displayed the inverse time-dependent change as compared to the miR-222 expression (Figure 2C, 3A). To verify whether miR-222 could suppress the endogenous PTEN level, we analyzed the effects of miR-222 transfection into DRG neurons on PTEN expression. qRT-PCR and Western blot analysis demonstrated that overexpression of miR-222 in DRG neurons suppressed the mRNA and protein levels of PTEN by about 50% as compared to control (Figure 3B). We further performed immunocytochemistry to map the cellular localization of PTEN in DRG neurons following transfection with miR-222 mimics, miR-222 inhibitor, or negative control (NC) at day 3 *in vitro* (DIV 3). The PTEN expression decreased in the soma of DRG neurons following transfection with miR-222 mimics; while the PTEN expression increased in the neurite end of DRG neurons following transfection with miR-222 inhibitor (Figure 3C). Our data suggested that miR-222 might probably regulate neurite growth through inhibiting the PTEN expression.

### Knockdown of PTEN Recapitulates the Effects of miR-222 on DRG Neurons

**Figure 4:**
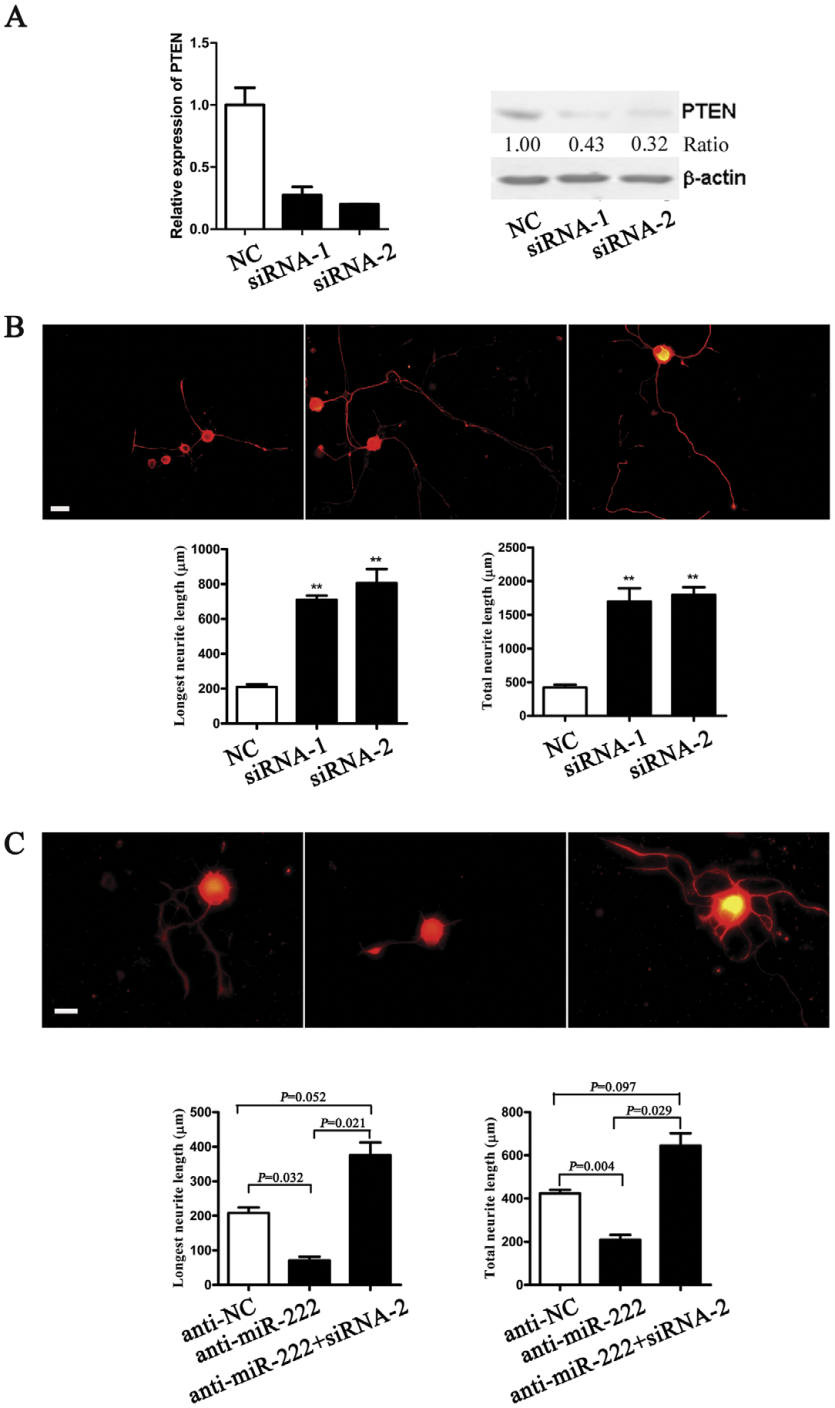
PTEN silencing reversed the suppressive effects of miR-222 inhibitor on DRG neurons. A. The mRNA or protein level of PTEN was down-regulated by transfection with PTEN-siRNA-1 (siRNA-1), or PTEN-siRNA-2 (siRNA-2) as compared with that of siRNA control (NC) in DRG neurons. B. Both siRNA-1 and siRNA-2 significantly promoted the neurite outgrowth from DRG neurons as compared with that of NC at 72 h after transfection. **P<0.01 vs. NC. Scale bar, 50 µm. C. The neurite outgrowth from DRG neurons was observed at 72 h after transfection with miR-222 inhibitor (anti-miR-222) with or without siRNA-2. Anti-miR-222 reduced the mean longest neurite length as well as the mean total neurite length (per neuron). In the presence of siRNA-2, anti-miR-222-mediated decrease in neurite outgrowth was abolished. Scale bar, 30 µm.

To explore the function of PTEN, two specific small interfering RNAs (siRNAs) against PTEN were synthesized. Both siRNA-1 and siRNA-2 remarkably reduced expression of PTEN at the mRNA and protein levels (Figure 4A). Neurite outgrowth assay showed that both siRNAs significantly facilitated the neurite growth of DRG neurons. Notably, siRNA-2 showed a more favorable effect than siRNA-1 because of the more effective knockdown of PTEN mRNA and protein by siRNA-2 (Figure 4B). It follows that knockdown of PTEN with siRNAs induces the effects on DRG neurons similar to those induced by miR-222.

Previous evidence shows that miR-222 post-transcriptionally regulates the PTEN expression by directly binding to the 3′-UTR of PTEN [27]. Based of our finding that PTEN expression showed down-regulation after nerve injury and thereby promoted neurite outgrowth from DRG neurons, we assumed that down-regulation of PTEN expression directly mediated miR-222-initiated neurite outgrowth from DRG neurons. To test this assumption, we performed transfection of miR-222 inhibitor into DRG neurons in the presence or absence of siRNA-2 against PTEN to decrease miR-222 expression. The results showed that there was a significant decrease in neurite outgrowth after transfected with miR-222 inhibitor alone, but a remarkable increase in the longest neurite length and total neurite length after co-transfected with miR-222 inhibitor and siRNA-2 against PTEN (Figure 4C), suggesting that PTEN might be a functional mediator for miR-222 in DRG neurons.

### miR-222 Regulates CREB Phosphorylation by Targeting PTEN to Promote Neurite Outgrowth from DRGs Neurons

**Figure 5:**
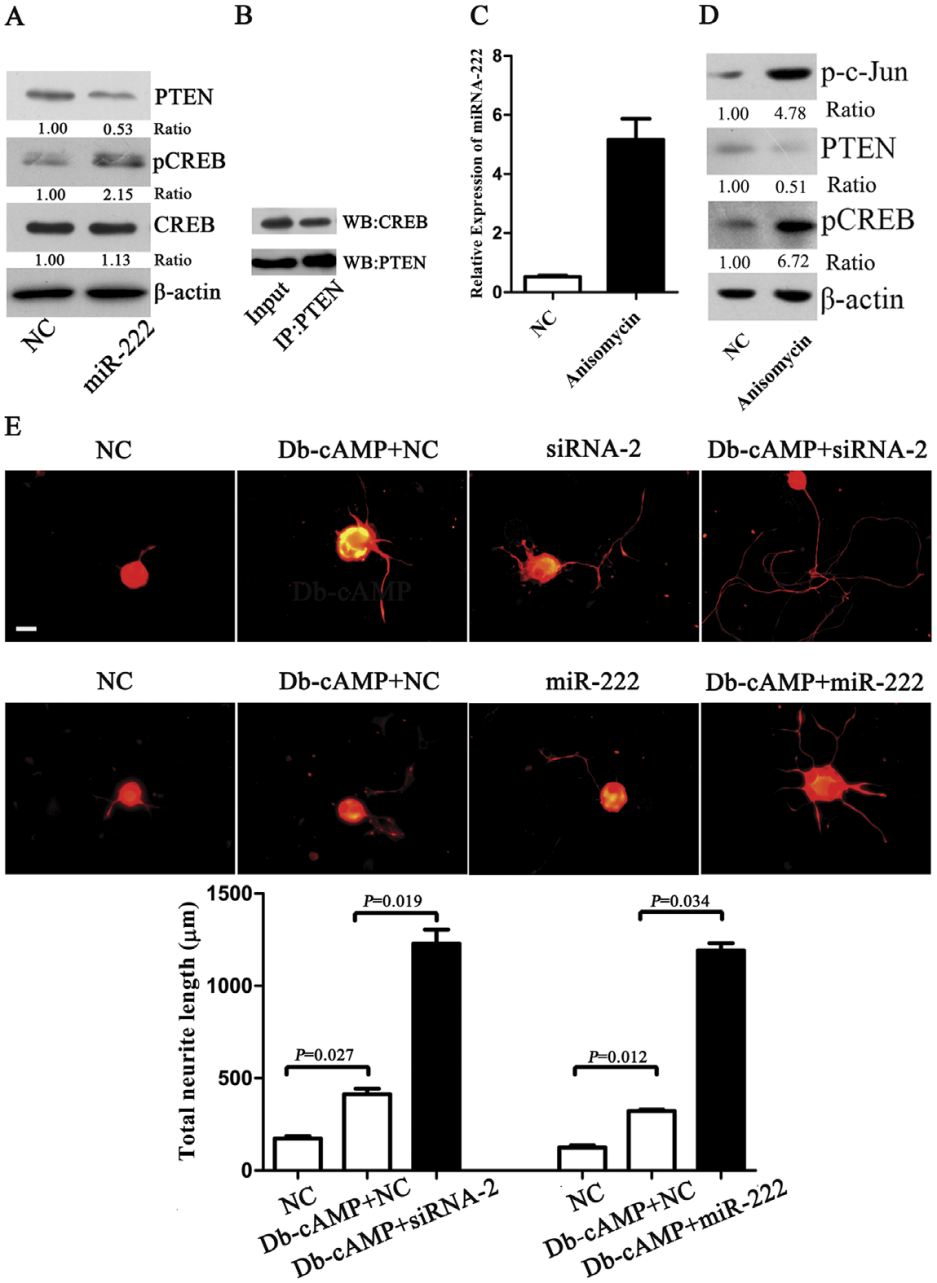
The effect of miR-222 on peripheral nerve regeneration. A. Impact of miR-222 on CREB phosphorylation in DRG neurons. Western blot analysis showed that the level of pCREB was up-regulated by transfection with miR-222 mimics (miR-222) as compared with that of mimics control (NC) in DRG neurons. B. Coimmunoprecipitation (IP) plus Western blotting (WB) of DRG neurons showed the interactions of PTEN with CREB. Input, total protein lysate blotted with anti-PTEN and anti-CREB antibodies. IP using PTEN (IP: PTEN) that pulled down both PTEN and CREB. WB using anti-PTEN and anti-CREB antibodies respectively (WB: PTEN and WB: CREB). C. qRT-PCR showed that the treatment with anisomycin (10 µM) for 30 min induced the miR-222 up-regulation in DRG neurons. D. Western blot analysis showed that treatment with anisomycin (10 µM) for 30 min induced PTEN down-regulation and pCREB up-regulation in DRG neurons. E. DRG neurons were fixed at 36 h after transfection, followed by neurite outgrowth assay. Knockdown of PTEN by either miR-222 or PTEN-siRNA-2 (siRNA-2) with addition of Db-cAMP (1 mM) for 20 min promoted the mean total neurite length (per DRG neuron) as compared to the stimulation of Db-cAMP alone. Scale bar, 30 µm.

We further examined the possible involvement of miR-222 in CREB phosphorylation. Western blot analysis demonstrated that pCREB level was increased in miR-222-overexpressing DRG neurons at DIV 3 (Figure 5A). Moreover, immunoprecipitation plus subsequent Western blotting showed that PTEN and CREB could be coimmunoprecipitated in cultured DRG neurons, thus contributing to our understanding of pCREB upregulation (Figure 5B). We also treated DRG neurons with anisomycin, an antibiotic routinely used to activate jun-N-terminal kinase that is responsible for c-Jun activation, and then determined the miR-222 and PTEN expression levels. qRT-PCR indicated that the miR-222 expression level significantly increased at 12 h after anisomycin treatment (Figure 5C); Western blot analysis revealed that the PTEN expression level decreased at 24 h after anisomycin treatment, and concurrently the pCREB expression level exhibited a significant increase (Figure 5D). In addition, knockdown of PTEN by either miR-222 or siRNA-2 against PTEN for 12 h plus the ensuing 20 min stimulation of dibutyryl-cAMP (Db-cAMP) promoted neurite outgrowth from DRG neurons as compared to the above-mentioned 12-h knockdown of PTEN or the stimulation of Db-cAMP alone (Figure 5E).

## Discussion

Contrary to the central nervous system (CNS), the peripheral nervous system (PNS) regenerates spontaneously after injury because of a permissive environment and activation of the intrinsic growth capacity of neurons. Peripheral nerve injury is accompanied by altered transcriptions of more than 1000 genes in DRGs [26], [29]. In this study, we investigated whether injury-induced miRNAs regulated the expression of these genes at the post-transcriptional level for conditioning the injured neurons into an actively growing state. The microarray-based analysis enabled us to identify 26 miRNAs in DRGs with significant expression alterations at different time points after nerve injury. Bioinformatics showed that the 26 miRNAs might be involved in many aspects of nerve regeneration, and provided an opportunity to decipher what target molecules of miRNAs could regulate nerve regeneration by integrating differentially expressed mRNAs with predicted targets. Out of 26 miRNAs, miR-223, 132 and 21 have been previously reported to be associated with nervous system. For example, miR-223 is highly expressed in neutrophils that are present in the spinal cord during the early phase of spinal cord injury [30]; miR-132 regulates dendritic spine morphology and synaptic physiology, contributes to the maturation of dendrites in newborn neurons in the adult hippocampus, and impacts the plasticity of visual cortex circuits [31], [32]; miR-21 is highly expressed in the spinal cord and DRGs following traumatic injury, thus promoting neurite outgrowth by down-regulating expression of Sprouty2 protein [18], [25]. Among the 26 miRNAs, we also investigated the functions of other miRNAs than miR-222, including miR-223, 132, 21, and 142-3p, and found that the latter 2 miRNAs had the promoting effect on neurite outgrowth from DRG neurons.

Using a bioinformatics approach, we analyzed potential targets of the differential expressed miRNAs post injury. GO enrichment analysis indicated that the 638 putative targets for these miRNAs were mainly involved in cell phenotype modulation-related function. These processes could play important roles in mobilizing the inherent capacity of neurite outgrowth and promoting nerve regeneration. The 638 putative targets were integrated with differentially expressed mRNA to acquire 38 potential targets, which, in particular, were associated with glutamine transport and sodium ion transport. These data were consistent with the knowledge that ion channels, transporters, and cell membrane receptors play essential roles in every step of nerve regeneration [33], and in agreement with the recent finding that release of glutamate from synaptic vesicles along axons of DRG neurons promotes myelin induction [34]. Anyway, injury-induced regulation of miRNAs may be beneficial to DRG neurons by these potential targets in the course of regeneration.

Very importantly, we observed for the first time the differentially expression of miR-222 in DRGs after nerve injury. miR-222 is a commonly dys-regulated miRNA in many forms of cancer [35], [36]; however its function in the nervous system has not been examined. miR-222 gene is located on chromosome Xp11.3, and functions as an oncogene by targeting the cell cycle inhibitor p27/Kip1, thereby controlling cell proliferation [37]-[39]. In addition, miR-222, by targeting PTEN and tissue inhibitor of metalloproteinases-3 (TIMP3) tumor suppressors, induces tumor necrosis factor (TNF)-related apoptosis-inducing ligand resistance and enhances cellular migration through activation of the Akt pathway and metallopeptidases [28], [40]. In this study, we observed that after nerve injury, miR-222 was up-regulated in DRGs until 14 days. This tendency of miR-222 up-regulation was matchable with the process of nerve regeneration, suggesting that miR-222 might trigger the intrinsic neurite growth from injured DRG neurons.

Several miRNAs, including miR-222 as well as miR-21 and miR-214, have been established as regulators of PTEN expression [41], [42]. Our data showed that miR-222 could suppress PTEN in DRG neurons, and so we postulated that the axotomy-induced increase in miR-222 expression decreased the PTEN protein level as to promote neurite outgrowth from injured DRG neurons. PTEN dephosphorylates PIP3, the second messenger produced by PI_3_K, and negatively regulates the activity of the serine/threonine protein kinase Akt [43]. Akt activation leads to phosphorylation and inhibition of tuberous sclerosis protein complex, which in turn results in subsequent engagement of Ras homolog enriched in brain to activate mTOR. PTEN attenuates regrowth of injured CNS axons by suppression of mTOR [9]. Importantly, different from the CNS regeneration, activation of the intrinsic growth capacity by PTEN inhibition in the PNS is independent of mTOR pathway [10]. A recent study has demonstrated that PTEN directly dephosphorylates CREB, and PTEN deficiency fails to affect the transcription of CREB but leads to pCREB upregulation independent of the PI_3_K/Akt pathway [44]. In this study, we demonstrated PTEN could interact with CREB in cultured DRG neurons, providing further evidence that CREB is a target of PTEN phosphatase and PTEN could directly dephosphorylate CREB. We also noted that PTEN increased CREB phosphorylation in DRG neurons, which might be responsible for the different mechanisms of nerve regeneration between CNS and PNS. In addition, knocked down of PTEN by endogenous miR-222 via c-Jun activation suggested that miR-222 might be an upstream regulator of PTEN inhibition during nerve regeneration.

Several studies suggest that elevation of intracellular cAMP is sufficient to initiate the neuronal intrinsic growth and promote regeneration of the central branch of DRG neurons after injury [5]. The effects of cAMP on regeneration seems to go through CREB [45], [46]. Peripheral nerve injury additionally induces c-Jun transcription factor-dependent regeneration-related gene expression. Conditional knockout of c-Jun gene expression in neurons reduces nerve regeneration [47]. As is known, c-Jun forms the activator protein-1 transcription factor, and is responsible for miR-222 activation in non small cell lung cancer and hepatocellular carcinoma [28]. In this study, we found that the pCREB level was increased in miR-222-overexpressing DRG neurons, and the miR-222 expression level was significantly increased by c-Jun activation. As is reported, some strategies are used to accelerate regenerative activity in the mature CNS. For instance, a combination of intraocular inflammation, cAMP elevation, and PTEN deletion results in unprecedented levels of regeneration following optic nerve injury. However, cAMP has little effect on its own and fails to enhance regeneration induced by PTEN deletion in the CNS [48]. Here, we attempted to examine whether increasing intracellular cAMP and inhibition of PTEN, with miR-222 or siRNA against PTEN, could exert synergistic effects on peripheral nerve regeneration. Our results showed that the above two ways seemed to exert complementary actions that enable cultured neurons to enhance regeneration. Collectively, all data suggest that miR-222 up-regulation inhibits PTEN, maintains CREB phosphorylation, and enlarges, at least in cultured neurons, the effects of cAMP.

In conclusion, on the basis of microarray profiling and functional analysis, we showed that miR-222, as one of the 26 differentiated miRNAs in DRG neurons after nerve injury, could regulate neurite growth by targeting PTEN, a major inhibitor of nerve regeneration. We also noted that knockdown of PTEN by miR-222 might be synergistic with cAMP/CREB-based signaling to improve the regenerative ability of neurons, at least in vitro.

## Materials and Methods

### Animal Surgery and Tissue Preparation

Thirty adult, male Sprague-Dawley (SD) rats (180–220 g) were randomly divided into five groups (n = 6 each) according to different time points of observation. After animals were anaesthetized by an intraperitoneal injection of complex narcotics, the sciatic nerve was exposed and lifted through an incision on the left lateral thigh. A 1 cm long sciatic nerve segment was resected at the site just proximal to the division of tibial and common peroneal nerves, and the incision sites were then closed. To minimize the discomfort and possible painful mechanical stimulation, the rats were housed in large cages with sawdust bedding after surgery. The L4-6 DRGs were harvested from different animal groups at 0, 1, 4, 7, and 14 d post axonomy, respectively. The animals in day 0 group underwent sham-surgery on the left sciatic nerve, serving as control. The experiment was repeated three times. All the experimental procedures involving animals were conducted in accordance with institutional animal care guidelines and approved ethically by the Administration Committee of Experimental Animals, Jiangsu Province, China.

### miRNA and mRNA Microarray

Total RNA was extracted using the mirVana™ miRNA Isolation Kit (Ambion, Austin, TX, USA) according to the manufacturer’s instructions. The quality of the purified RNA was assessed by a BioAnalyzer 2100 (Agilent Technologies, Santa Clara, CA, USA). The purified RNA was quantified by determining the absorbance at 260 nm with a Nanodrop ND-1000 spectrophotometer (Infinigen Biotechnology Inc., City of Industry, CA). A miRNA microarray (Agilent Technology), containing probes for the complete Sanger miRBase 10.0, was used to screen RNA from rat DRGs of different groups. The labeling and hybridization were performed at the Shanghai Biochip Company (Shanghai, China), according to the protocols in the Agilent miRNA microarray system. Agilent Scan Control software was used for scanning the microarray slides, and Agilent Feature Extraction software version 9.5.3 was used for image analysis. Microarray data were analyzed using GeneSpring GX v11.0 software (Agilent Technology). Both mRNA and miRNA expression were profiled with the idential RNA samples by using the microarray.

### Bioinformatics Analysis

miRNA and mRNA expression profiles were scanned by the microarray after segmentation of the peripheral nerve. Two groups of data were compared, the significance and false discovery rate were calculated using the adjusted F-test with the Random Variation Model [49], and the differentially expressed genes in these serial time points were obtained via screening.

Using the miRNAs with significant expression variance, we conducted the following analysis: 1) Hierarchical clustering with the expression of these miRNAs. We calculated Z-score from the expression of miRNA. The euclidean distance measure was used to compute the distance (dissimilarity) in both ways (miRNA and time). 2) Searching for putative targets of miRNA using TargetScan and miRanda database, the intersection of these two datasets was used as the prediction results of the target genes of these differentially expressed miRNAs, and then integrating putative miRNA targets with differentially expressed mRNA yielding potential targets. 3) GO analyses for the integrated targets. In detail, two-sided Fisher’s exact test was used to classify the GO category, and the type I error was calculated to correct the *P* value.

### Cell Culture and Oligonucleotide Transfection

DRG neurons were dissociated and maintained *in vitro* using a modification from the method [10]. Briefly, the L4–L6 DRGs were removed from the adult male SD rats (180–220 g) and transferred to Ca^2+^/Mg^2+^-free Hank’s buffered salt solution (CMF-HBSS), where the axon roots and dural tissue were manually removed. The DRGs were rinsed three times in CMF-HBSS and then transferred to a tube containing 2 ml of 0.1% collagenase type I (Sigma, St louis, MO). Following a 90 min incubation at 37°C, DRG were incubated in 0.25% trypsin (Invitrogen, Carlsbad, CA) for an additional 25 min at 37°C. The DRGs were placed into single-cell suspension by triturating 10–15 times every 7 min through 1 ml pipette tips, and the single-cell suspension was spun for 5 min at 800 rpm at 4°C. After the spin, the cells were resuspended and passed through a 70 µm mesh, and then placed in 500 µl of L15 medium enriched with 1∶100 dilution of N2 supplement (Invitrogen) and 0.1% BSA (Sigma) and placed into a culture medium of DMEM/F12 (Invitrogen) + 1∶100 dilution of N2, 0.5% BSA, and 0.2 ng/ml NGF (RD Systems, Inc. Tustin, CA) plus 50 U of penicillin/ml, 50 U streptomycin/ml (Invitrogen) and plated onto poly-L-lysine (Sigma) and 10 µg/ml laminin (Invitrogen)-coated plates.

To carry out the neurite growth assay with randomly sampled neurons, the transfection efficiency must be nearly 100%. To achieve this, at the time of plating, neurons were transfected with miRNA mimics, miRNA inhibitor, or siRNAs (Ribobio, Guangzhou, China) respectively using Lipofectamine RNAiMAX reverse transfection reagent (Invitrogen), according to the manufacturer’s instructions in couple with low-density (500-800 cell per 1 cm^2^) plating as previously stated. After being cultured in transfection reagent overnight (12 h), neurons were transferred to fresh culture medium to allow incubation for additional 60 h or 24 h.

### Immunocytochemistry

DRG cultures were fixed for 30 min with 4% paraformaldehyde at room temperature, and then rinsed three times for 5 min each with PBS. The cells were permeabilized for 10 min in 0.1% Triton X-100 (Sigma), then rinsed twice for 5 min in PBS, and then blocked for 1 h in PBS with 5% BSA at room temperature. Then anti-PTEN antibody (1∶200, Santa Cruz Biotechnology, Santa Cruz, CA), anti-NF-200 antibody (1∶200, sigma) was incubated with the neurons at 4°C for 24 h. After rinse three times with PBS, neurons were reacted with FITC goat anti-mouse IgG (1∶200, sigma) and Cy3 sheep anti-rabbit IgG (1∶400, sigma) at room temperature for 2 h. Then cells were stained with Hoechst 33342 for 5 min.

### *In situ* Hybridization

*In situ* hybridization was performed using the miRCURY LNA™ microRNA ISH Optimization Kit (Exiqon, VedBaek, Denmark) according to the manufacturer’s instructions. Each section was treated with 3 mg/ml proteinase K for 20 min at 37°C. After treatment with 0.2% glycine-PBS for 5 min, sections were washed 2 times in PBS for 5 min each and acetylated with 0.25% acetic anhydride in 0.1 M triethanolamine hydrochloride for 10 min. Hybridization with DIG-labeled probes against miR-222 was carried out for 2 hours at 55°C in hybridization buffer. Scrambled probes were used as a control. After hybridization, sections were washed in 5× SSC for 5 min at 55°C, 1× SSC 2 times for 5 min at 55°C, 0.2× SSC 2 times for 5 min at 55°C, and 0.2× SSC for 5 min at room temperature. Blocking was performed for 2 hours at room temperature with alkaline phosphatase-conjugated Fab anti-DIG antibody (Roche, Mannheim, Germany) in 2% sheep serum. The slides were stained using 5-bromo-4-chloro-3-indolyl-phosphate and nitroblue tetrazolium (Roche), and then counter stained with Nuclear Fast Red™ (Vector Labs, Burlingame, CA).

### Neurite Outgrowth Assay

The neurite outgrowth assay was performed as described previously [10]. Neurons were transfected with miRNA mimics, miRNA inhibitor or siRNAs at DIV 0. At DIV 3 (72 h), neurons were fixed in 4% paraformaldehyde and subjected to immunocytochemistry with anti-NF-200 (Sigma) [29]. Cultured neurons were subjected to transfection by either miR-222 or siRNA-2 for 12 h and then treated with or without Db-cAMP (1 mM, 20 min), followed by 24-h incubation in plain medium and fixation by 4% paraformaldehyde [5], [50], Neurite outgrowth analyses were carried out in quadruplicate. Neurites were observed under a DMR fluorescent microscope (Leica Microsystems, Bensheim, Germany) with representative photomicrographs being captured throughout. The longest neurite length and the total (summed) length of all neurites for each of the first 200 neurons encountered during scanning (regardless of size and number of neurites) was measured with Leica QWin V3 image analysis program.

### qRT-PCR

Reverse-transcribed complementary DNA was synthesized with the Prime-Script RT reagent Kit (TaKaRa, Dalian, China). PCR was performed with SYBR Premix Ex Taq (TaKaRa). For miRNA detection, mature miR-222 was reverse-transcribed with specific RT primers, quantified with a TaqMan probe, and normalized by RNU6B mature miRNA using TaqMan miRNA assays (Applied Biosystems, Foster City, CA). The relative expression level was calculated using the comparative 2^-ΔCt^ method.

### Western Blot Analysis

Protein extracts were prepared from cultured cells or DRG tissues. Equal amounts of protein were subjected to SDS-PAGE and electrotransferred to PVDF membrane (Bio-Rad, Hercules, CA). The membrane was blocked with 5% nonfat dry milk in Tris-HCl buffered saline, pH 7.4 with Tween-20 and incubated with the primary antibodies against PTEN (1∶1000, Cell Signaling Technology, Beverly, MA), CREB (1∶1000, Cell Signaling Technology), pCREB (Ser133, 1∶1000, Cell Signaling Technology), phosphorylated c-Jun (p-c-Jun) (Ser63, 1∶1000, Santa Cruz Biotechnology), respectively, overnight at 4°C. The β-actin (1∶5000, sigma) served as a loading control. Then the HRP-conjugated species-specific secondary antibody (Santa Cruz Biotechnology) was added and measured by an enhanced chemiluminescence kit (Pierce Chemical Company, Rockford, IL).

### Coimmunoprecipitation

The cultured neurons from rat L4–L6 DRGs were lysed in a lysis buffer (0.025 M Tris, 0.15 M NaCl, 0.001 M EDTA, 1% NP-40, and 5% glycerol, pH 7.4) containing protease and phosphatase inhibitors. The immunoprecipitation was performed according to the guides of the Pierce® Classic IP Kit (Pierce). Afterwards, cell samples were subjected to Western blot analysis through the above mentioned procedures but using primary antibodies against PTEN (1∶1000, Rabbit, Cell Signaling Technology), and CREB (1∶500, mouse, Abcam, Cambridge, UK) instead.

### Statistical Analysis

All data were expressed as means ± S.D, and the Student’s *t* test was used for comparison between groups. Statistical analyses were done by means of SPSS 15.0 for windows (SPSS, Chicago, IL). *P*<0.05 was considered statistically significant.
