# Correction: microRNA-222 Targeting PTEN Promotes Neurite Outgrowth from Adult Dorsal Root Ganglion Neurons following Sciatic Nerve Transection

**DOI:** 10.1371/annotation/ffd17f66-3f4e-4119-ac58-bfa4b0c50912

**Published:** 2012-09-17

**Authors:** Songlin Zhou, Dingding Shen, Yongjun Wang, Leilei Gong, Xiaoyan Tang, Bin Yu, Xiaosong Gu, Fei Ding

There was an error in the published funding statement. The correct funding is:

This study was supported by National Natural Science Foundation of China (Grant Nos. 81130080, 81171180 and 31200799), and the Priority Academic Program Development of Jiangsu Higher Education Institutions (PAPD). The funders had no role in study design, data collection and analysis, decision to publish, or preparation of the manuscript. 

